# Crank fore-aft position alters the distribution of work over the push and pull phase during synchronous recumbent handcycling of able-bodied participants

**DOI:** 10.1371/journal.pone.0220943

**Published:** 2019-08-19

**Authors:** Riemer J. K. Vegter, Barry S. Mason, Bastiaan Sporrel, Benjamin Stone, Lucas H. V. van der Woude, Vicky L. Goosey-Tolfrey

**Affiliations:** 1 Department of Human Movement Sciences, University Medical Center Groningen, University of Groningen, Groningen, The Netherlands; 2 Peter Harrison Centre for Disability Sport, School of Sport, Exercise & Health Sciences, Loughborough University, Loughborough, United Kingdom; 3 Center for Rehabilitation, University Medical Center Groningen, University of Groningen, Groningen, The Netherlands; Norwegian University of Science and Technology, NORWAY

## Abstract

**Objective:**

The objective of the current study was to investigate the effect of four different crank fore-aft positions on elbow flexion and shoulder protraction, the consequent propulsion kinetics and the physiological responses during handcycling.

**Methods:**

Twelve able-bodied male participants volunteered in this study. Crank fore-aft positions were standardised at 94%, 97%, 100% and 103% of the participants’ arm length. Two submaximal 3 min trials were performed at a fixed cadence (70 rpm), in a recumbent handcyle attached to an ergometer at two fixed power outputs (30W and 60W). Elbow flexion, shoulder protraction, propulsion kinetics and physiological responses of the participants were continuously measured.

**Results:**

As crank fore-aft distance increased, a decrease in elbow flexion (42±4, 37±3, 33±3, 29±3°) and an increase shoulder protraction was observed (29±5, 31±5, 34±5, 36±5°). The percentage of work done in the pull phase increased as well (62±7, 65±7, 67±6, 69±8%, at 60W), which was in line with an increased peak torque during the pull phase (8.8±1.6, 9.0±1.4, 9.4±1.5, 9.7±1.4Nm, at 60W) and reduced peak torque during the push phase (6.0±0.9, 5.6±0.9,5.6±0.9, 5.4±1.0Nm, in 60W condition). Despite these changes in work distribution, there were no significant changes in gross mechanical efficiency (15.7±0.8, 16.2±1.1, 15.8±0.9, 15.6±1.0%, at 60W). The same patterns were observed in the 30W condition.

**Conclusions:**

From a biomechanical perspective the crank position closest to the trunk (94%) seems to be advantageous, because it evens the load over the push and pull phase, which reduces speed fluctuations, without causing an increase in whole body energy expenditure and hence a decrease of gross mechanical efficiency. These findings may help handcyclists to optimize their recumbent handcycle configuration.

## Introduction

Recumbent handcycles are used for recreational sports and have been further optimized for two Paralympic sports, i.e. handcycling and paratriathlon [1]. These typically synchronously operated handcycles should be individually configured towards an athlete, for an optimal athletic performance in terms of power transfer, injury prevention and reductions of drag resistance. That said, little research has been done on how to individually configure the handcycle to the athlete. Currently, the only restriction imposed by the Union Cycliste Internationale (UCI) is that the crank height must be lower than the eye-line of the handcyclist [2]. Therefore, numerous areas of the handcycle-user interface can be manipulated, such as the backrest inclination [3–5], crank length [5–7] or handgrip orientation [7,8]. A recent qualitative study with expert handcyclists and coaches identified the horizontal displacement of the crank-axis with respect to the athlete, known as the crank fore-aft position, to be a key area of handcycle configuration with regards to performance [9]. A parallel can be made with wheelchair seat-height studies that similarly looked at the axle position with respect to the upper body, which showed that gross mechanical efficiency at a given task-load can be optimized through changes in wheelchair configuration [10,11].

When exploring the effects of handcycling configuration, both the biomechanical and the physiological effects are relevant with regard to performance [12]. The propulsion cycle consists of a synchronous push and pulling phase of both upper limbs [7]. From a biomechanical perspective an even distribution of work throughout both phases will reduce forward speed fluctuations and thus might be preferred, as this reduces the external power output demands at a constant speed [13] and leads to lower peak forces [14], which in turn is thought to reduce the risk of overuse injuries [15]. From a physiological perspective, handcycling under steady-state conditions can be evaluated by calculating the gross mechanical efficiency, i.e. the ratio of work accomplished with respect to the energy expended [16] and has been used as a relevant outcome measure for different handcycling optimisation studies [12,17]. Thus, the crank fore-aft position that leads to a more even work distribution and a higher gross mechanical efficiency under a given power output and forward speed, might be considered most beneficial to the athlete.

Previous studies have looked at the effect of changing the crank fore-aft position in a number of different experimental setups, with a great diversity in the number of participants, types of handcycles used and subsequent recommendations. Two studies used able-bodied participants and an arm crank ergometer; the first with an asynchronous crank setup (thus dissimilar to handcycling) found an increase in oxygen uptake during a maximal test to exhaustion at the crank position closest to the participant (30° elbow flexion, 0° being full extension) [18]. The second study used a synchronous arm crank setup and tested submaximal conditions, showing that the 30° elbow flexion also had a higher gross mechanical efficiency compared to the 15° elbow flexion [19]. A more recent study on crank fore-aft position was performed with people with a spinal cord injury in an actual force-instrumented handcycle, yet this was an add-on device, attached to a wheelchair for activities of daily living, rather than an athletic recumbent handcycle [4]. By use of this setup no physiological improvements were found for a closer crank fore-aft position, while they did show an increase of the load on several rotator cuff muscles in the closer crank fore-aft position[4].

Interpretation of the aforementioned findings for athletic recumbent handcycling is further made difficult because a standardised definition of crank fore-aft positions is currently lacking. Although handcycling can be considered a closed chain activity, there are still multiple degrees of freedom, especially in the combination of elbow flexion/extension with the amount of shoulder protraction. Hence, both might be influenced when changing the crank fore-aft position and therefore should both be considered [20]. Yet, previous studies used elbow flexion only as standardisation criteria for the crank fore-aft distance [4,18,19], overlooking the other degrees of freedom. In the current study we will standardize the crank fore-aft position to arm-length and consequently have the individual combination of elbow flexion and shoulder protraction as a result, possibly differing among individuals and/or crank conditions.

Subsequently the objective of the current study was to investigate the effect of four different crank fore-aft positions on 1) the resulting elbow flexion/extension and shoulder protraction angles, 2) the consequent propulsion kinetics (torque profile around the crank-axis), and 3) the physiological responses (VO2, gross mechanical efficiency and heartrate). Based on the previous work on efficiency in arm crank ergometry and other handcycles, a closer crank fore-aft position is hypothesized to be more efficient [3,18,19].

## Methods

### Participants

Twelve able-bodied male participants with little or no previous experience of handcycling volunteered for this study (age 25±5 years; body mass 76±8 kg; height 1.82±0.05 m; arm length 0.68±0.02 m). All participants provided written informed consent after receiving written and verbal information about the protocol and objectives of the study. The study was performed in accordance with the declaration of Helsinki and approved by the local university ethics committee (Loughborough University, reference number R16-P060).

### Experimental protocol

**Fig 1:**
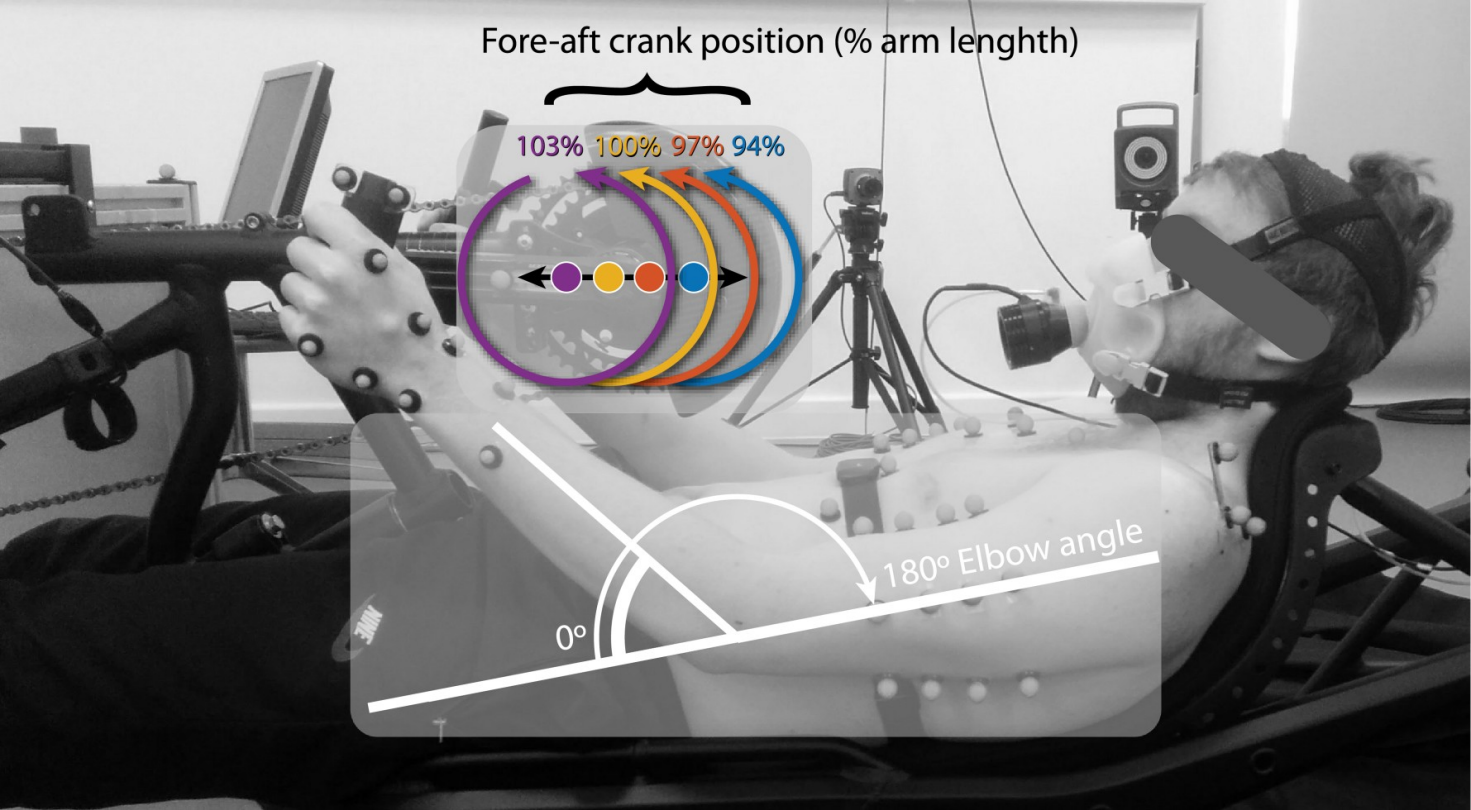
A recumbent handcyclist, in the experimental setup. Four different crank fore-aft configurations were tested, which were standardised at 94%, 97%, 100% and 103% of participants arm length. Elbow flexion was defined as zero in full extension and increased positively when flexed. Shoulder protraction was defined as the angle of the scapula with regard to the y-axis of the local coordinate system of the thorax, where zero meant a parallel position of the two [21].

Trials were conducted in a custom made, fully adjustable handcycle (Fig 1, Schmicking Reha-Technik GmbH, Holzwickede, Germany), which was attached to an ergometer (Cyclus 2, Richter, Germany). The configuration of the handcycle was standardised to a 170 mm crank length, a 15° (pronated) handgrip angle and a crank height allowing 20 mm of clearance between the abdomen and handgrip (lowest position). Based on pilot testing the maximal manipulation range (i.e. the minimum & maximum crank fore-aft) of the crank fore-aft position was determined so that the participants would still be able to access the cranks with adequate clearance throughout the whole cycle. Four appropriate crank fore-aft configurations were chosen, which were standardised to achieve a 94%, 97%, 100% and 103% distance of the participants’ arm length, as seated in the recumbent handcycle whilst having the crank handle rotated the furthest away from the body. 94% Armlength was found to be the feasible minimum, without the gearing system hitting the chest, while 103% was found to be the maximum whilst keeping the participants’ back to the backrest. Arm length was measured from the acromial angle to the distal end of the fifth metacarpal, while the participant kept their elbow extended with the palms facing towards the side of the body. Crank fore-aft positions were measured from the acromial angle to the centre of the handle, while the pedal was in a horizontal position pointing away from the participant. During these measurements, the participants were asked to sit in a relaxed position with their hands on their lap to keep their posture constant while configuring the handcycle.

Participants cycled in each of the four configurations at a fixed power output of 30W and 60W [4,19], resulting in a total of eight different trials. Each trial lasted three minutes, of which the final minute was used for analysis, followed by a four-minute rest. Configurations were performed in a counterbalanced order, with the high and low PO conditions alternating to reduce the effect of fatigue. During all trials, participants were asked to maintain a constant cadence of 70rpm using a visual display, since changes in crank rate affect oxygen consumption [22], and could therefore confound the results. A cadence of 70rpm was chosen because it was found to be physiologically suitable for arm-cranking [23] and has been used in several other studies on handcycling [4,19,22].

Able-bodied participants were chosen to counteract the training effect of athletes that have used one setup for a long time, making them prefer a certain mode. Since the participants were inexperienced in handcycling, two familiarisation sessions consisting of the whole protocol were conducted in the two weeks prior to the measurement session. The two familiarisation trials followed the same protocol as the measurement session. The practice-order of the crank fore-aft positions was counterbalanced over all participants, to prevent bias because of a training effect.

### Elbow flexion & shoulder protraction

Upper-body 3D kinematics were collected at 100Hz, with a passive marker motion capture system (Vicon Motion Systems, Oxford, UK). Fifty-five reflective markers were placed on the participants: nine were placed on the thorax (two on the lower ribs, xiphoid process, incisura jugular, C7, T8 and a cluster of three markers on the middle part of the sternum); twelve were placed on each upper arm (four anterior, four lateral and four posterior), four on each forearm (ulnar and radial styloid and on the proximal part of the ulna and radius), four on each hand (proximal and distal end of the second and fifth metacarpal bones), and a cluster of three markers was placed bilaterally on the acromion [24]. The last two markers were placed bilaterally on the cranks. Before starting the experiment, a number of calibrations were recorded to determine the locations of anatomical landmarks and the joint centre of the glenohumeral joint [25]. Thereafter, the C7 and T8 markers were replaced with virtual markers (in respect to the thorax cluster) during the handcycling trials due to marker occlusion caused by the participant’s recumbent position [9,20]. The identified landmarks were; the sternoclavicular joint, the acromioclavicular joint, the acromion angle, trigonium spinae, the inferior angle of the scapula, and the medial and lateral epicondyle of the humerus. All landmarks were identified on both the left and right side of the participants. After determining these landmarks one trial was recorded in which the participant was asked to do circumduction movements for ten seconds. This trial was then used to determine the glenohumeral joint centre using the Symmetrical Centre of Rotation Estimation (SCoRE) (Ehrig et al., 2005), necessary for calculating the elbow flexion angle.

Raw kinematic data was first analysed using the Vicon Nexus software package (version 2.5), and was further analysed using custom written Matlab scripts (Matlab 2015, Mathworks, Inc). Euler angles for all segments were calculated following the International Society of Biomechanics recommendations [21]. The elbow flexion and shoulder protraction angle for each crank fore-aft position were calculated at the furthest crank-handle distance during each cycle, and averaged over all cycles within the last minute of a trial. Elbow flexion was defined as zero in full extension and increased positively when flexed. Shoulder protraction was defined according to the recommendation of the International Society of Biomechanics as the angle of the scapula with regard to the y-axis of the local coordinate system of the clavicle, where zero meant a parallel position of the two [21].

### Kinetics

**Fig 2:**
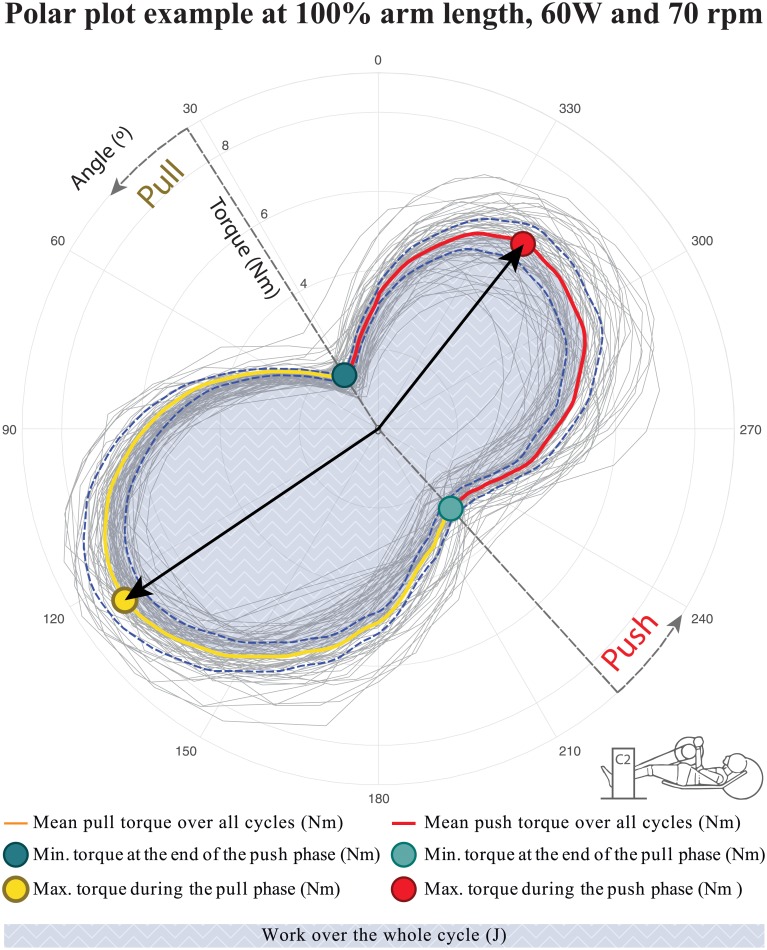
Definition of the kinetic variables based on the in-plane torque around the instrumented crank data over all cycles from a single trial of a single participant. C2 stands for Cyclus2 (Handcycle ergometer).

Cycle kinetics were measured with an instrumented crank (‘Rotor Impower 3D+’, Rotor Bike Components, Ajalvir, Spain) which measured the effective torques exerted around the crank-axis, combined with the angle of the crank. Different kinetic parameters were calculated as descriptors for force distribution over the cycle: minimal torque at the end of the push- and pull phase, maximal torque in the push and pull phase, and percentage of work done in the pull phase (Fig 2). The push phase was defined from the minimum torque closest to the participant, to the minimum torque furthest away. Minimum and maximum torque of the phases were extracted from the torque profile and averaged over all cycles in the last minute of each trial for each participant. Percentage of work done in the pull phase was calculated by dividing the work done in the pull phase by the total work done (x100%). The total work (Nm) delivered was calculated for both the push- and pull phase by taking the product of angular velocity and exerted torque and integrating this over time.

### Physiology

Oxygen uptake (VO_2_) and carbon dioxide (CO_2_) were continuously measured with a breath-by-breath gas analyser (Cortex Metalyzer 3B, Cortex, Leipzig, Germany), that also included a heart rate monitor for synchronized heartrate measurements (polar RS400, Kempele, Finland). Before each session the gas analyser was calibrated according to the manufacturer’s recommendations. Data collected over the last minute of each exercise trial were averaged and taken to reflect physiological steady-state handcycling. From the VO_2_ (L/min), and respiratory exchange ratio (RER) the energy expenditure (EE) was determined (formula 1, [26].

$$EE\;(W)\;=\;\frac{(4,94∙RER\;+16,04)∙VO₂}{60}$$ (1)

The gross mechanical efficiency was calculated as the ratio of power output (30W or 60W performed on the Cyclus2) over energy expenditure:

$$GME\;(\%)\;=\;\frac{Pₒ}{EE}\;∙100$$ (2)

### Statistical analyses

All statistical analyses were done using the Statistical Package for Social Sciences (SPSS version 23.0; IBM corp., Armonk, NY, USA). Two-way repeated measures analyses of variance (ANOVA) were performed to explore differences in kinematics, kinetics and physiological parameters between crank fore-aft positions (94–103%) and power output (30/60W). Significance level was set at p < 0.05. Mauchly’s test of sphericity was performed prior to conducting the ANOVA to test if the assumption of sphericity was met. When a significant effect was found, post hoc contrasts were performed to determine where the differences occurred (with Bonferroni correction). For the minimal torque at the end of the push phase a difference contrast was used, where the mean of each configuration was compared to the mean of the previous configuration. For the percentage of work done a simple contrast was used where the mean of each configuration was compared to the 94% configuration.

## Results

All participants were able to complete the protocol while maintaining a steady cadence of 70 rpm in each condition. Only the kinematic data of participant 5 in the 100% condition was not recorded properly.

### Elbow flexion & shoulder protraction

The measured arm length, crank position difference between the closest and furthest configurations (manipulation range), and average elbow extension and shoulder protraction angles in each configuration are shown for each participant in Table 1. For each increasing crank fore-aft position the repeated measures anova (p<0.001) and contrasts showed that elbow flexion decreased (42±4, 37±3, 33±3, 29±3° (mean±SD)) and shoulder protraction increased (29±5, 31±5, 34±5, 36±5°). Moreover, between participants different elbow flexion angles (range: 35–48, 32–43, 28–38, 25–34) and shoulder protraction angles (range: 20–37, 21–39, 24–43, 29–46) were chosen to accommodate the same relative crank fore-aft position.

**Table 1:** Arm length, manipulation range and mean elbow flexion (mean±SD) and shoulder protraction angles (mean±SD) at maximal extension for each participant in each configuration (at 30W & 60W).

participant	Armlength	Manipulation	Elbow flexion at maximal extension (°)				Shoulder protraction at maximal extension (°)			
(nr)	(cm)	range (cm)	94%	97%	100%	103%	94%	97%	100%	103%
1	66.8	6.5	35±1.1	35±1.1	33±1.2	29±1.2	31±0.9	31±1.2	35±0.8	36±0.6
2	70.9	6.9	38±1.4	32±1.9	29±1.3	26±1.8	27±1.3	26±1.2	29±1.2	30±0.9
3	70.2	6.5	39±2	36±2	32±1.4	28±1.3	37±1.3	39±1.1	41±1.4	42±1.3
4	69.7	7.3	40±1.6	36±1.4	28±1.2	25±1.3	30±1.4	31±1.0	33±0.9	34±1.6
5	66.9	6	41±1.2	37±1.4	-	30±1.7	23±0.9	26±1.4	-	31±0.7
6	64.2	5.5	41±1.7	38±1.9	32±1.6	32±2.8	31±1.1	32±1.2	33±1.4	35±1.3
7	66.6	7.8	42±0.6	32±0.4	30±1	26±1.1	26±0.8	29±0.9	30±0.9	35±0.6
8	66.5	5.5	42±0.7	40±0.9	38±0.6	34±1.2	35±0.6	37±1.4	38±1.0	39±1.0
9	69.7	7.4	43±0.9	37±2.6	32±1.6	29±1.4	28±1.6	30±1.2	33±1.1	38±1.5
10	68.7	7	47±2	42±2	35±1.9	33±1.3	20±1.6	21±1.7	24±0.5	29±2.0
11	70.1	8	47±1.2	40±1.2	36±1.5	27±1.8	29±1.0	31±1.0	35±1.4	37±0.7
12	67.2	8.5	48±1.2	43±2.4	36±2.2	34±1.7	36±0.8	39±2.1	43±1.6	46±1.1
**Mean±SD**	**68.1±2.0**	**6.9±0.96**	**42±4**	**37±3**	**33±3**	**29±3**	**29±5.1**	**31±5.4**	**34±5.4**	**36±4.9**

### Kinetics

**Fig 3:**
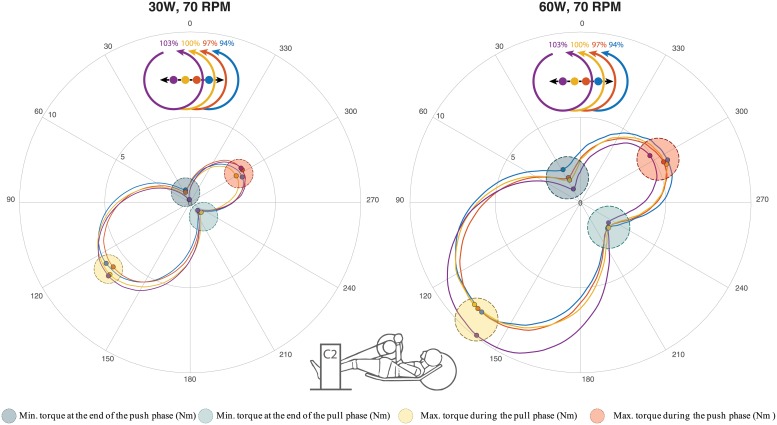
Torque profiles per crank fore-aft position, averaged over all full cycles over the last minute and all participants in the 30W (left) and 60W (right) conditions. C2 stands for Cyclus2 (Handcycle ergometer). Contrasts revealed that the minimal torque at the end of the push phase decreased, the maximal torque in the pull phase increased and the percentage of work done in pull phase increased when the crank-fore-aft position increased.

The kinetic variables are shown in Table 2. The mean torque profiles of the different configurations, averaged over all full cycles in the last minute and consequently over all participants, are shown in Fig 3. Contrasts revealed that for the minimal torque at the end of the push phase (torque end push), each configuration resulted in significantly lower torque at the end of the push phase compared to the previous (94% vs 97%: p = 0.001; 97% vs 100%: p < 0.001; 100% vs 103%: p < 0.001). Contrasts for the maximal torque in the pull phase (peak pull) showed that all but the first two configurations differed significantly from each other (94% vs 97%: p = 0.606; 97% vs 100%: p = 0.005; 100% vs 103%: p = 0.007). Contrasts also showed that the percentage of work done in the pull phase was significantly lower in the 94% configuration compared to the the 100% and 103% configurations (p = 0.003; p = 0.020), but not lower compared to the 97% configurations (p = 0.164).

**Table 2:** Means and standard deviations of all analysed dependent variables and results of the two-way repeated measures Anova.

Variables		94%	97%	100%	103%	P-Configuration	p-Power	P-Interaction
peak push (N/m)	30W	3.5±1.0	3.5±0.8	3.2±0.8	3.5±1.0	0.153	0.000	0.381
	60W	6.0±0.9	5.6±0.9	5.6±0.9	5.4±1.0			
peak pull (N/m)	30W	6.2±1.4	6.2±1.2	6.4±1.2	6.5±1.3	0.002	0.000	0.196
	60W	8.8±1.6	9.0±1.4	9.4±1.5	9.7±1.4			
torque end push (N/m)	30W	0.7±0.5	0.5±0.4	0.2±0.4	0.2±0.3	0.000	0.000	0.000
	60W	2.0±0.6	1.6±0.5	1.4±0.5	1.0±0.5			
torque end pull (N/m)	30W	0.7±0.3	0.7±0.3	0.7±0.4	0.7±0.4	0.334	0.000	0.202
	60W	1.8±0.7	2.0±0.6	1.8±0.6	1.9±0.6			
Work done in pull phase (%)	30W	69±12	70±10	73±10	70±11	0.003	0.043	0.165
	60W	62±7	65±7	67±6	69±8			
VO2 (L/min)	30W	0.78±0.07	0.78±0.07	0.79±0.06	0.8±0.04	0.17	0.000	0.626
	60W	1.08±0.06	1.05±0.09	1.08±0.07	1.1±0.09			
HR (beats/min)	30W	90±13	88±12	87±13	89±12	0.341	0.000	0.808
	60W	105±13	103±13	103±13	104±12			
Gross Mechanical Efficiency (%)	30W	11.2±0.9	11.2±0.9	11.1±0.7	11.9±0.5	0.051	0.000	0.413
	60W	15.7±0.8	16.2±1	15.8±0.9	15.6±1			

### Physiology

Test results for VO_2_, ME and HR are shown in Table 2 as well. The repeated measures analysis showed that VO_2_, ME and HR were not significantly affected by the configurations. On the other hand the higher power output of the 60W condition did lead to a higher VO_2,_ GME and HR compared to the 30W condition.

## Discussion

This is the first study to examine the biomechanical and physiological effects of different crank fore-aft positions during recumbent handcycling of able-bodied participants relative to arm length. The increase in crank fore-aft position resulted in a decrease in elbow flexion and an increase in shoulder protraction. Consequently, the distribution of work shifted towards an increase of work being performed in the pull phase and a decrease of work being performed in the push phase over the propulsion cycle. This shift was concomitant with an increased peak torque during the pull phase and a reduced peak torque during the push phase. Despite these clear changes in work distribution between the push and pull phase, there were no significant changes in physiological responses at the same power output, as hypothesised based on previous literature.

A novel approach to standardize crank fore-aft position was implemented to define changes in crank fore-aft position relative to the participants’ arm length. This allowed for an abundant number of combinations of shoulder protraction and elbow flexion to help rotate the crank of the handcycle. Indeed, from the results it becomes clear that both elbow flexion and shoulder protraction are used to accommodate the different experimental crank fore-aft positions. For the whole group both a decrease in elbow flexion and an increase of shoulder protraction was present for each increase in crank fore-aft position. Moreover, at an individual level, the chosen combination of angles differed considerably between participants. Participants found different solutions and/or have different preferences with regard to the task, which might also be dependent on upper-body physical fitness and motor learning of this relatively new task [27]. This emphasizes the importance to not standardize crank fore-aft position based on elbow flexion angle only, as reported previously [18,19], since this would lead to inconsistent changes with respect to the amount of shoulder protraction between individuals.

Next to the increased elbow flexion and decreased shoulder protraction in the closer crank fore-aft positions, the participants also increased the work done in the push phase. Since participants were performing at a fixed cadence and power output this meant they consequently reduced the work done in the pull phase to maintain a constant power output. Such a more equal distribution of work is generally preferred in handcycle races [13], as this is thought to distribute the work over multiple muscle groups, thereby delaying fatigue of individual muscle groups. Thus, a closer crank fore-aft position is preferred from this standpoint. Moreover, handcycling is performed with a synchronous crank-setup, where the hands rotate in-phase, instead of the 180° phase difference in leg-cycling. Thus, having a more continuous power output reduces forward speed fluctuations, which is beneficial in real life conditions because such fluctuations require additional power output to maintain an average velocity, since power output is dependent on the square of the velocity [28].

Yet, the changes in crank fore-aft position will also have influenced the movement pattern and muscle mechanics in terms of length-force and force-velocity characteristics around the elbow and shoulder joint [10,11,29,30]. Yet, the relationships between pedal force and crank angular velocity in cycling were shown to be less curved than the intrinsic force–velocity relationship of muscles would suggest [31] Given the difficult multi-joint dynamics and the lack of change in gross mechanical efficiency it is hard to define the optimal upper-body movement pattern based on the current study. Currently, little is known about an optimal propulsion technique for handcyclists even though some biomechanical studies have tried to better understand the actual force production effectiveness, using different approaches (FeF, PFPI) [32–34]. Parameters such as the Fraction of Effective Force (FeF) or the Postural Force Production Index (PFPI) might help understand how the upper-body performs optimal work throughout the propulsion cycle. Studies combining such approaches with the measurement of actual handcycling athletes are necessary to better understand what optimal handcycling crank setups and athletic propulsion technique are and which muscle groups are best used to perform work while handcycling.

Although a closer crank fore-aft position led to a different kinetic profile, no such effects were found for the physiological variables of VO_2_, ME or HR. This was similar to the results of Arnet et al. 2014, who also couldn’t identify the best crank fore-aft position based on physiological measurements [4]. An earlier study did find a significant, albeit very small increase for gross mechanical efficiency for an elbow flexion of 30° vs 15° during synchronous arm cranking of able-bodied participants at 35W (8.02±1.44% vs 7.98±1.41%) [19]. Their small effect was attributed to the low PO that they used and consequently they expected a larger effect at a higher PO, which was not confirmed by the present study. Since the participants performed the same workload for each condition, not finding an effect on ME might indicate that there is no clear energetical advantage of predominantly performing work in the pull phase in the most extended crank fore-aft position over a more equal distribution of work between the pull and push phase in the closest one.

The results of the 60W condition showed RER values slightly above one, indicating an anaerobic component to the participants’ work, indicative of a non-steady-state workload. Previous work with able-bodied participants were also confronted with this problem, but at much higher workloads [4,19,22,35]. For instance, Verellen et al [35] tested able-bodied participants in a synchronous mode at 130W (65 rpm) and also reported RER’s slightly over one. However, they argued that despite the consequent possible overestimation of gross mechanical efficiency due to the anaerobic component of power production, there was little reason to believe this was different between the tested conditions (arm power vs arm-trunk power) which holds similarly for the crank fore-aft positions of the current study.

Based on the results of the current study different recommendations to athletic handcyclists in a recumbent handcycle can be made, in light of several possible limitations. We showed the effect of crank fore-aft position in a group of relatively untrained able-bodied individuals in a static lab-setup without steering or forward-backward oscillations, highlighting the changes in work distribution. Athletic handcyclists on the other hand have different impairments and have to manoeuvre themselves outside during a race [36]. The limited power output possible by the relatively inexperienced able-bodied participants can be considered the biggest limitation in the translation of the findings to actual athletic handcyclists. More knowledge on the scaling of the torque production curve in dependence of higher power outputs is currently not available for actual handcyclists. However, another study with able-bodied participants did show a proportional change in the torque production curve over a range of 20-120W [20].

Despite these differences we still expect the following to be of relevance. First, the crank fore-aft position does not only influence elbow flexion, but also shoulder protraction. Therefore the possibility to produce work by shoulder pro/re traction is also expected to be present in athletic handcyclists and needs to be investigated further [15,37]. Second, the crank fore-aft position matters for the distribution of work over the push and pull cycle. Different arguments can be made as to why a closer crank fore-aft position would be advantageous, but this will also be dependent on the individual athletes’ capacity and/or upper body impairment. To that end the novel experimental set-up developed in the current study could be used to individually tailor the handcycle dimensions better to an athlete, by experimentally changing the crank fore-aft position. Moreover, we advise to further explore the full upper-body kinematics including the range of motion of the elbow flexion and shoulder protraction angle. The hypotheses would be that athletes would benefit similarly from having the crank fore-aft position closer to their body. Finally, from a physiological perspective no difference in overall expended energy was found, indicating that there might be some freedom of choice, without directly impacting whole body energetics.

To optimize athletic handcycle performance there is a need to better understand what the ideal work distribution over the propulsion cycle is with regard to multiple outcomes, such as optimal power production, injury risk and local fatigue of muscles. Currently, multiple instrumented crank systems are available with a high sample rate of the exerted torque. The systematic use of such devices during competition, training and carefully controlled lab experiments have the potential to further our understanding about optimal cyclic upper body exercise and creating an optimal interface between the athlete and the handcycle. Moreover, we encourage the relatively little population of high performance handcyclists to share data about their handcycle setup and current choice of crank fore-aft position, to further the sport as a whole and help with the general understanding about optimal fit and performance, which will help the professionalisation of the sport in the future.

## Conclusion

The current study revealed that an increase in crank fore-aft position resulted in a decreased elbow flexion and increased shoulder protraction during recumbent handcycling which led to an increase of work being performed in the pull phase and a decrease of work being performed in the push phase, without changing gross mechanical efficiency. In line with our hypothesis, the closest position possible for the athlete resulted in a more even distribution of work, because it evens the load over the pull and push phase and reduces forward speed fluctuations. Contrary to our expectations no changes in whole body energy expenditure were found. These findings in able-bodied participants helps build theory on optimal handcycle configuration, to be applied and tested in in actual elite handcyclists with a broad range of impairments.

## Supporting information

S1 File SPSS Dataset of the performed statistics. (SAV)
